# Supplementary material for: Registered report protocol: A scoping review to identify potential predictors as features for developing automated estimation of the probability of being frail in secondary care
Source: PLoS One. 2022 Sep 27;17(9):e0275230. doi: 10.1371/journal.pone.0275230 (PMC9514620; doi:10.1371/journal.pone.0275230)
Supplement: S2 Appendix — (DOCX) [file pone.0275230.s002.docx]

**S2 Appendix - Pubmed (MEDLINE) search**

**Date: 31-AUG-2022**

**Building block 1**

MeSH

| “Frail Elderly”[Mesh] | 14.167 |
| --- | --- |
| “Frailty”[Mesh] | 7.216 |
| “Functional status”[Mesh] | 1.109 |

Synonyms:

| “Frail*”[tiab] | 31.818 |
| --- | --- |
| “Debilit*”[tiab] | 31.029 |
| “Geriatric syndrome*”[tiab] | 1.931 |

Free terms:

| “Pre-frail*”[tiab] | 1.277 |
| --- | --- |
| “Functional status”[tiab] | 28.885 |
| “Fragil*”[tiab] | 49.798 |
| “Vulnerab*”[tiab] | 172.278 |

Possible antonyms:

| “Resilien*”[tiab] | 51.159 |
| --- | --- |

| (“Frail Elderly”[Mesh] **OR** “Frailty”[Mesh] **OR** “Functional status”[Mesh] **OR** “Frail*”[tiab] **OR** “Debilit*”[tiab] **OR** “geriatric syndrome*”[tiab] **OR** “Pre-frail*”[tiab] **OR** “Functional status”[tiab] **OR** “Fragil*”[tiab] **OR** “Vulnerab*”[tiab] **OR** “Resilien*”[tiab]) | 358.061 |
| --- | --- |

**Building block 2**

MeSH

| “Sensitivity and Specificity”[Mesh] | 639.089 |
| --- | --- |
| “Risk Assessment”[Mesh] | 305.234 |
| “Surveys and Questionnaires”[Mesh] | 1.180.438 |

Free terms

| “tool*”[tiab] | 903.568 |
| --- | --- |
| “instrument*”[tiab] | 333.212 |
| “predictive model*”[tiab] | 27.435 |
| “prediction model*”[tiab] | 30.870 |
| “questionnaire*”[tiab] | 621.906 |
| “Risk assessment”[tiab] | 83.538 |
| “index*”[tiab] | 1.004.966 |
| “inventor*”[tiab] | 102.516 |
| “survey*”[tiab] | 785.745 |
| “assessment method*”[tiab] | 17.332 |

| (“Risk Assessment”[Mesh] **OR** “Surveys and Questionnaires”[Mesh] **OR** “tool*”[tiab] **OR** “instrument*”[tiab] **OR** “predictive model*” [tiab] **OR** “prediction model*”[tiab] **OR** “questionnaire*”[tiab] **OR** “Risk assessment”[tiab] **OR** “index*”[tiab] **OR** “inventor*”[tiab] **OR** “survey*”[tiab] **OR** “assessment method*”[tiab]) | 4.067.600 |
| --- | --- |

**Building block 3**

MeSH

| “Aged”[Mesh] | 3.412.346 |
| --- | --- |
| “Geriatrics”[Mesh] | 31.203 |

Free terms

| “Aged”[tiab] | 682.388 |
| --- | --- |
| “Elde*”[tiab] | 297.089 |
| “Olde*”[tiab] | 536.728 |
| “geriatric*”[tiab] | 73.694 |
| “centenarian*”[tiab] | 2.318 |
| “centarian*”[tiab] | 3 |
| “nonagenarian*”[tiab] | 1.674 |
| “octogenarian*”[tiab] | 3.831 |
| “octagenarian*”[tiab] | 53 |
| “septuagenarian*”[tiab] | 463 |
| “very old”[tiab] | 4.767 |
| “senior*”[tiab] | 48.555 |

| (“Aged”[Mesh] **OR** “Geriatrics”[Mesh] **OR** “Aged”[tiab] **OR** “Elde*”[tiab] **OR** “Olde*”[tiab] **OR** “geriatric*”[tiab] **OR** “centenarian*”[tiab] **OR** “centarian*”[tiab] **OR** “nonagenarian*”[tiab] **OR** “octogenarian*”[tiab] **OR** “octagenarian*”[tiab] **OR** “septuagenarian*”[tiab] **OR** “very old”[tiab] **OR** “senior*”[tiab]) | 4.193.155 |
| --- | --- |

**Building block 4**

Free terms

| “risk factor*”[tiab] | 717.489 |
| --- | --- |
| “variable*”[tiab] | 947.570 |
| “predictor*”[tiab] | 463.601 |
| “parameter*”[tiab] | 1.194.464 |
| “deficit*”[tiab] | 279.057 |
| “characteristic*”[tiab] | 1.681.532 |
| “determinant*”[tiab] | 273.390 |
| “criteri*”[tiab] | 766.686 |

| (“risk factor*”[tiab] OR “variable*”[tiab] OR “predictor*”[tiab] OR “parameter*”[tiab] OR “deficit*”[tiab] OR “characteristic*”[tiab] OR “determinant*”[tiab] OR “criteri*”[tiab]) | 5.265.974 |
| --- | --- |

**Building blocks combined (exploratory search, date: 31-AUG-2021)**

| ((“Frail Elderly”[Mesh] **OR** “Frailty”[Mesh] **OR** “Functional status”[Mesh] **OR** “Frail*”[tiab] **OR** “Debilit*”[tiab] **OR** “geriatric syndrome*”[tiab] **OR** “Pre-frail*”[tiab] **OR** “Functional status” [tiab] **OR** “Fragil*”[tiab] **OR** “Vulnerab*”[tiab] **OR** “Resilien*”[tiab]) **AND** (“Risk Assessment”[Mesh] **OR** “Surveys and Questionnaires”[Mesh] **OR** “tool*”[tiab] **OR** “instrument*”[tiab] **OR** “predictive model*”[tiab] **OR** “prediction model*”[tiab] **OR** “questionnaire*”[tiab] **OR** “Risk assessment” [tiab] **OR** “index*”[tiab] **OR** “inventor*”[tiab] **OR** “survey*”[tiab] **OR** “assessment method*”[tiab]) **AND** (“Aged”[Mesh] **OR** “Geriatrics”[Mesh] **OR** “Aged”[tiab] **OR** “Elde*”[tiab] **OR** “Olde*”[tiab] **OR** “geriatric*”[tiab] **OR** “centenarian*”[tiab] **OR** “centarian*”[tiab] **OR** “nonagenarian*”[tiab] **OR** “octogenarian*”[tiab] **OR** “octagenarian*”[tiab] **OR** “septuagenarian*”[tiab] **OR** “very old”[tiab] **OR** “senior*”[tiab]) **AND** (“risk factor*”[tiab] **OR** “variable*”[tiab] **OR** “predictor*”[tiab] **OR** “parameter*”[tiab] **OR** “deficit*”[tiab] **OR** “characteristic*”[tiab] **OR** “determinant*”[tiab] **OR** “criteri*”[tiab])) | 20.757 (no filters used) |
| --- | --- |
| (((“Frail Elderly”[Mesh] **OR** “Frailty”[Mesh] **OR** “Functional status”[Mesh] **OR** “Frail*”[tiab] **OR** “Debilit*”[tiab] **OR** “geriatric syndrome*”[tiab] **OR** “Pre-frail*”[tiab] **OR** “Functional status” [tiab] **OR** “Fragil*”[tiab] **OR** “Vulnerab*”[tiab] **OR** “Resilien*”[tiab]) **AND** (“Risk Assessment”[Mesh] **OR** “Surveys and Questionnaires”[Mesh] **OR** “tool*”[tiab] **OR** “instrument*”[tiab] **OR** “predictive model*”[tiab] **OR** “prediction model*”[tiab] **OR** “questionnaire*”[tiab] **OR** “Risk assessment” [tiab] **OR** “index*”[tiab] **OR** “inventor*”[tiab] **OR** “survey*”[tiab] **OR** “assessment method*”[tiab]) **AND** (“Aged”[Mesh] **OR** “Geriatrics”[Mesh] **OR** “Aged”[tiab] **OR** “Elde*”[tiab] **OR** “Olde*”[tiab] **OR** “geriatric*”[tiab] **OR** “centenarian*”[tiab] **OR** “centarian*”[tiab] **OR** “nonagenarian*”[tiab] **OR** “octogenarian*”[tiab] **OR** “octagenarian*”[tiab] **OR** “septuagenarian*”[tiab] **OR** “very old”[tiab] **OR** “senior*”[tiab]) **AND** (“risk factor*”[tiab] **OR** “variable*”[tiab] **OR** “predictor*”[tiab] **OR** “parameter*”[tiab] **OR** “deficit*”[tiab] **OR** “characteristic*”[tiab] **OR** “determinant*”[tiab] **OR** “criteri*”[tiab]) **AND** (dutch[la] **OR** english[la]) **AND** (aged[filter]) **AND** (2018:2020[pdat])) **OR** ((“Frail Elderly”[Mesh] **OR** “Frailty”[Mesh] **OR** “Functional status”[Mesh] **OR** “Frail*”[tiab] **OR** “Debilit*”[tiab] **OR** “geriatric syndrome*”[tiab] **OR** “Pre-frail*”[tiab] **OR** “Functional status” [tiab] **OR** “Fragil*”[tiab] **OR** “Vulnerab*”[tiab] **OR** “Resilien*”[tiab]) **AND** (“Risk Assessment”[Mesh] **OR** “Surveys and Questionnaires”[Mesh] **OR** “tool*”[tiab] **OR** “instrument*”[tiab] **OR** “predictive model*”[tiab] **OR** “prediction model*”[tiab] **OR** “questionnaire*”[tiab] **OR** “Risk assessment” [tiab] **OR** “index*”[tiab] **OR** “inventor*”[tiab] **OR** “survey*”[tiab] **OR** “assessment method*”[tiab]) **AND** (“Aged”[Mesh] **OR** “Geriatrics”[Mesh] **OR** “Aged”[tiab] **OR** “Elde*”[tiab] **OR** “Olde*”[tiab] **OR** “geriatric*”[tiab] **OR** “centenarian*”[tiab] **OR** “centarian*”[tiab] **OR** “nonagenarian*”[tiab] **OR** “octogenarian*”[tiab] **OR** “octagenarian*”[tiab] **OR** “septuagenarian*”[tiab] **OR** “very old”[tiab] **OR** “senior*”[tiab]) **AND** (“risk factor*”[tiab] **OR** “variable*”[tiab] **OR** “predictor*”[tiab] **OR** “parameter*”[tiab] **OR** “deficit*”[tiab] **OR** “characteristic*”[tiab] **OR** “determinant*”[tiab] **OR** “criteri*”[tiab]) **AND** (dutch[la] **OR** english[la]) **AND** (2021:2022[pdat]))) | 7.796 (including approximately 495 reviews identified using filters: Meta-Analysis, Review, Systematic Review) |

**Inclusion criteria:**

1. Topic of article = Frailty

**Exclusion criteria:**

1. Frailty is the independent variable for another outcome and there is no description of independent variables predicting frailty. (Rationale: fits goal to find independent variables to predict frailty.)(e.g. Research article describing how much frailty predicts complications or mortality without describing which variables predict frailty = exclusion)
2. Article type: case studies are excluded. (Rationale: we consider case studies not suitable for providing information on potential predictor variables.)
3. Full text not available in English / Dutch language (Rationale: readability, no full text article = no complete review possible.)
