# Supplementary material for: Registered report protocol: A scoping review to identify potential predictors as features for developing automated estimation of the probability of being frail in secondary care
Source: PLoS One. 2022 Sep 27;17(9):e0275230. doi: 10.1371/journal.pone.0275230 (PMC9514620; doi:10.1371/journal.pone.0275230)
Supplement: S3 Appendix — (DOCX) [file pone.0275230.s003.docx]

**S3 Appendix - CINAHL Plus, Embase, and Web of Science search**

**CINAHL Plus**

**Date: 30-AUG-2022**

**Building block 1**

MH

| MH “Frail Elderly” | 8.524 |
| --- | --- |

Synonyms:

| TI “frail*” OR AB “frail*” | 16.796 |
| --- | --- |
| TI “debilit*” OR AB “debilit*” | 8.696 |
| TI “geriatric syndrome*” OR AB “geriatric syndrome*” | 973 |

Free terms:

| TI “pre-frail*” OR AB “pre-frail*” | 657 |
| --- | --- |
| TI “functional status” OR AB “functional status” | 11.552 |
| TI “fragil*” OR AB “fragil*” | 9.254 |
| TI “vulnerab*” OR AB “vulnerab*” | 59.704 |

Possible antonyms:

| TI “resilien*” OR AB “resilien*” | 20.588 |
| --- | --- |

| MH “Frail Elderly” **OR** TI “frail*” OR AB “frail*” **OR** TI “debilit*” OR AB “debilit*” **OR** “TI geriatric syndrome*” OR AB “geriatric syndrome*” **OR** TI “pre-frail*” OR AB “pre-frail*” **OR** TI “functional status” OR AB “functional status” **OR** TI “fragil*” OR AB “fragil*” **OR** TI “vulnerab*” OR AB “vulnerab*” **OR** TI “resilien*” OR AB “resilien*” | 124.938 |
| --- | --- |

**Building block 2**

MH

| MH “Risk Assessment” | 134.513 |
| --- | --- |
| MH “Research Instruments+” | 1.221.083 |
| MH “Surveys” | 159.088 |
| MH “Survey Research” | 21.480 |

Free terms

| TI “tool*” OR AB “tool*” | 216.312 |
| --- | --- |
| TI “instrument*” OR AB “instrument*” | 106.889 |
| TI “predictive model*” OR AB “predictive model*” | 5.992 |
| TI “prediction model*” OR AB “prediction model*” | 7.871 |
| TI “questionnaire*” OR AB “questionnaire*” | 262.949 |
| TI “Risk assessment” OR AB “Risk assessment” | 18.353 |
| TI “index*” OR AB “index*” | 261.641 |
| TI “inventor*” OR AB “inventor*” | 42.206 |
| TI “survey*” OR AB “survey*” | 322.327 |
| TI “assessment method*” OR AB “assessment method*” | 5.957 |

| MH “Risk Assessment” **OR** MH “Research Instruments+” **OR** MH “Surveys” **OR**  MH “Survey Research”  **OR** TI “tool*” OR AB “tool*” **OR** TI “instrument*” OR AB “instrument*” **OR** TI “predictive model*” OR AB “predictive model*” **OR** TI “prediction model*” OR AB “prediction model*” **OR**  TI “questionnaire*” OR AB “questionnaire*” **OR** TI “Risk assessment” OR AB “Risk assessment” **OR** TI “index*” OR AB “index*” **OR** TI “inventor*” OR AB “inventor*” **OR** TI “survey*” OR AB “survey*” **OR** TI “assessment method*” OR AB “assessment method*” | 1.880.941 |
| --- | --- |

**Building block 3**

MH

| MH “Geriatrics” | 6.750 |
| --- | --- |

Free terms

| TI “Aged” OR AB “Aged” | 226.345 |
| --- | --- |
| TI “Elde*” OR AB “Elde*” | 115.577 |
| TI “Olde*” OR AB “Olde*” | 239.374 |
| TI “geriatric*” OR AB “geriatric*” | 29.399 |
| TI “centenarian*” OR AB “centenarian*” | 798 |
| TI “centarian*” OR AB “centarian*” | 2 |
| TI “nonagenarian*” OR AB “nonagenarian*” | 655 |
| TI “octogenarian*” OR AB “octogenarian*” | 1.176 |
| TI “octagenarian*” OR AB “octagenarian*” | 14 |
| TI “septuagenarian*” OR AB “septuagenarian*” | 133 |
| TI “very old” OR AB “very old” | 1.615 |
| TI “senior*” OR AB “senior*” | 28.871 |

| MH “Geriatrics” **OR** TI “Aged” OR AB “Aged” **OR** TI “Elde*” OR AB “Elde*” **OR** TI “Olde*” OR AB “Olde*” **OR** TI “geriatric*” OR AB “geriatric*” **OR** TI “centenarian*” OR AB “centenarian*” **OR** TI “centarian*” OR AB “centarian*” **OR** TI “nonagenarian*” OR AB “nonagenarian*” **OR** TI “octogenarian*” OR AB “octogenarian*” **OR** TI “octagenarian*” OR AB “octagenarian*” **OR** TI “septuagenarian*” OR AB “septuagenarian*” **OR** TI “very old” OR AB “very old” **OR** TI “senior*” OR AB “senior*” | 530.376 |
| --- | --- |

**Building block 4**

Free terms

| TI “risk factor*” OR AB “risk factor*” | 215.369 |
| --- | --- |
| TI “variable*” OR AB “variable*” | 240.394 |
| TI “predictor*” OR AB “predictor*” | 162.971 |
| TI “parameter*” OR AB “parameter*” | 145.639 |
| TI “deficit*” OR AB “deficit*” | 65.624 |
| TI “characteristic*” OR AB “characteristic*” | 318.878 |
| TI “determinant*” OR AB “determinant*” | 57.892 |
| TI “criteri*” OR AB “criteri*” | 217.684 |

| TI “risk factor*” OR AB “risk factor*” **OR** TI “variable*” OR AB “variable*” **OR** TI “predictor*” OR AB “predictor*” **OR** TI “parameter*” OR AB “parameter*” **OR** TI “deficit*” OR AB “deficit*” **OR** TI “characteristic*” OR AB “characteristic*” **OR** TI “determinant*” OR AB “determinant*” **OR** TI “criteri*” OR AB “criteri*” | 1.135.439 |
| --- | --- |

**Building blocks combined (exploratory search, date: 30-AUG-2022)**

| (MH “Frail Elderly” **OR** TI “frail*” OR AB “frail*” **OR** TI “debilit*” OR AB “debilit*” **OR** TI “geriatric syndrome*” OR AB “geriatric syndrome*” **OR** TI “pre-frail*” OR AB “pre-frail*” **OR** TI “functional status” OR AB “functional status” **OR** TI “fragil*” OR AB “fragil*” **OR** TI “vulnerab*” OR AB “vulnerab*” **OR** TI “resilien*” OR AB “resilien*”) **AND** (MH “Risk Assessment” **OR** MH “Research Instruments+” **OR** MH “Surveys” **OR** MH “Survey Research”  **OR** TI “tool*” OR AB “tool*” **OR** TI “instrument*” OR AB “instrument*” **OR** TI “predictive model*” OR AB “predictive model*” **OR** TI “prediction model*” OR AB “prediction model*” **OR** TI “questionnaire*” OR AB “questionnaire*” **OR** TI “Risk assessment” OR AB “Risk assessment” **OR** TI “index*” OR AB “index*” **OR** TI “inventor*” OR AB “inventor*” **OR** TI “survey*” OR AB “survey*” **OR** TI “assessment method*” OR AB “assessment method*”) **AND** (MH “Geriatrics” **OR** TI “Aged” OR AB “Aged” **OR** TI “Elde*” OR AB “Elde*” **OR** TI “Olde*” OR AB “Olde*” **OR** TI “geriatric*” OR AB “geriatric*” **OR** TI “centenarian*” OR AB “centenarian*” **OR** TI “centarian*” OR AB “centarian*” **OR** TI “nonagenarian*” OR AB “nonagenarian*” **OR** TI “octogenarian*” OR AB “octogenarian*” **OR** TI “octagenarian*” OR AB “octagenarian*” **OR** TI “septuagenarian*” OR AB “septuagenarian*” **OR** TI “very old” OR AB “very old” **OR** TI “senior*” OR AB “senior*”) **AND** (TI “risk factor*” OR AB “risk factor*” **OR** TI “variable*” OR AB “variable*” **OR** TI “predictor*” OR AB “predictor*” **OR** TI “parameter*” OR AB “parameter*” **OR** TI “deficit*” OR AB “deficit*” **OR** TI “characteristic*” OR AB “characteristic*” **OR** TI “determinant*” OR AB “determinant*” **OR** TI “criteri*” OR AB “criteri*”) | 8.974  (no filters used) |
| --- | --- |
| (MH “Frail Elderly” **OR** TI “frail*” OR AB “frail*” **OR** TI “debilit*” OR AB “debilit*” **OR** TI “geriatric syndrome*” OR AB “geriatric syndrome*” **OR** TI “pre-frail*” OR AB “pre-frail*” **OR** TI “functional status” OR AB “functional status” **OR** TI “fragil*” OR AB “fragil*” **OR** TI “vulnerab*” OR AB “vulnerab*” **OR** TI “resilien*” OR AB “resilien*”) **AND** (MH “Risk Assessment” **OR** MH “Research Instruments+” **OR** MH “Surveys” **OR** MH “Survey Research”  **OR** TI “tool*” OR AB “tool*” **OR** TI “instrument*” OR AB “instrument*” **OR** TI “predictive model*” OR AB “predictive model*” **OR** TI “prediction model*” OR AB “prediction model*” **OR** TI “questionnaire*” OR AB “questionnaire*” **OR** TI “Risk assessment” OR AB “Risk assessment” **OR** TI “index*” OR AB “index*” **OR** TI “inventor*” OR AB “inventor*” **OR** TI “survey*” OR AB “survey*” **OR** TI “assessment method*” OR AB “assessment method*”) **AND** (MH “Geriatrics” **OR** TI “Aged” OR AB “Aged” **OR** TI “Elde*” OR AB “Elde*” **OR** TI “Olde*” OR AB “Olde*” **OR** TI “geriatric*” OR AB “geriatric*” **OR** TI “centenarian*” OR AB “centenarian*” **OR** TI “centarian*” OR AB “centarian*” **OR** TI “nonagenarian*” OR AB “nonagenarian*” **OR** TI “octogenarian*” OR AB “octogenarian*” **OR** TI “octagenarian*” OR AB “octagenarian*” **OR** TI “septuagenarian*” OR AB “septuagenarian*” **OR** TI “very old” OR AB “very old” **OR** TI “senior*” OR AB “senior*”) **AND** (TI “risk factor*” OR AB “risk factor*” **OR** TI “variable*” OR AB “variable*” **OR** TI “predictor*” OR AB “predictor*” **OR** TI “parameter*” OR AB “parameter*” **OR** TI “deficit*” OR AB “deficit*” **OR** TI “characteristic*” OR AB “characteristic*” **OR** TI “determinant*” OR AB “determinant*” **OR** TI “criteri*” OR AB “criteri*”) **filter: English language, Dutch/Flemish language; Publication Year: 2021-2022** | 1.691 (including approximately 104 reviews identified using filters: Meta-Analysis, Meta Synthesis Review, Systematic Review) |
| (MH “Frail Elderly” **OR** TI “frail*” OR AB “frail*” **OR** TI “debilit*” OR AB “debilit*” **OR** TI “geriatric syndrome*” OR AB “geriatric syndrome*” **OR** TI “pre-frail*” OR AB “pre-frail*” **OR** TI “functional status” OR AB “functional status” **OR** TI “fragil*” OR AB “fragil*” **OR** TI “vulnerab*” OR AB “vulnerab*” **OR** TI “resilien*” OR AB “resilien*”) **AND** (MH “Risk Assessment” **OR** MH “Research Instruments+” **OR** MH “Surveys” **OR** MH “Survey Research”  **OR** TI “tool*” OR AB “tool*” **OR** TI “instrument*” OR AB “instrument*” **OR** TI “predictive model*” OR AB “predictive model*” **OR** TI “prediction model*” OR AB “prediction model*” **OR** TI “questionnaire*” OR AB “questionnaire*” **OR** TI “Risk assessment” OR AB “Risk assessment” **OR** TI “index*” OR AB “index*” **OR** TI “inventor*” OR AB “inventor*” **OR** TI “survey*” OR AB “survey*” **OR** TI “assessment method*” OR AB “assessment method*”) **AND** (MH “Geriatrics” **OR** TI “Aged” OR AB “Aged” **OR** TI “Elde*” OR AB “Elde*” **OR** TI “Olde*” OR AB “Olde*” **OR** TI “geriatric*” OR AB “geriatric*” **OR** TI “centenarian*” OR AB “centenarian*” **OR** TI “centarian*” OR AB “centarian*” **OR** TI “nonagenarian*” OR AB “nonagenarian*” **OR** TI “octogenarian*” OR AB “octogenarian*” **OR** TI “octagenarian*” OR AB “octagenarian*” **OR** TI “septuagenarian*” OR AB “septuagenarian*” **OR** TI “very old” OR AB “very old” **OR** TI “senior*” OR AB “senior*”) **AND** (TI “risk factor*” OR AB “risk factor*” **OR** TI “variable*” OR AB “variable*” **OR** TI “predictor*” OR AB “predictor*” **OR** TI “parameter*” OR AB “parameter*” **OR** TI “deficit*” OR AB “deficit*” **OR** TI “characteristic*” OR AB “characteristic*” **OR** TI “determinant*” OR AB “determinant*” **OR** TI “criteri*” OR AB “criteri*”) **filter: English language, Dutch/Flemish language; Age 65+, 80+; Publication Year: 2018-2020** | 1.031 (including approximately 58 reviews identified using filters: Meta-Analysis, Review, Systematic Review) |

**Inclusion criteria:**

1. Topic of article = Frailty

**Exclusion criteria:**

1. Frailty is the independent variable for another outcome and there is no description of independent variables predicting frailty. (Rationale: fits goal to find independent variables to predict frailty.)(e.g. Research article describing how much frailty predicts complications or mortality without describing which variables predict frailty = exclusion)
2. Article type: case studies are excluded. (Rationale: we consider case studies not suitable for providing information on potential predictor variables.)
3. Full text not available in English / Dutch language (Rationale: readability, no full text article = no complete review possible.)

**Embase**

**Date: 27-May-2022**

**Building block 1**

Emtree

| 1 | exp frail elderly/ | 11.469 |
| --- | --- | --- |
| 2 | exp frailty/ | 19.624 |
| 3 | exp functional status/ | 61.893 |

Synonyms:

| 4 | Frail* .ti. OR Frail* .ab. | 44.623 |
| --- | --- | --- |
| 5 | Debilit* .ti. OR Debilit* .ab. | 44.364 |
| 6 | "geriatric syndrome*".ti. OR "geriatric syndrome*".ab | 3.153 |

Free terms:

| 7 | pre-frail* .ti. OR pre-frail* .ab. | 1.976 |
| --- | --- | --- |
| 8 | “functional status” .ti. OR “functional status” .ab. | 42.597 |
| 9 | fragil* .ti. OR fragil* .ab. | 62.141 |
| 10 | vulnerab* .ti. OR vulnerab* .ab. | 211.786 |

Possible antonyms:

| 11 | resilien* .ti. OR resilien* .ab. | 52.718 |
| --- | --- | --- |

| 12 | 1 or 2 or 3 or 4 or 5 or 6 or 7 or 8 or 9 or 10 or 11 | 487.584 |
| --- | --- | --- |

**Building block 2**

Emtree

| 13 | exp Risk Assessment/ | 666.298 |
| --- | --- | --- |
| 14 | exp questionnaire/ or exp health survey/ | 1.039.148 |

Free terms

| 15 | tool* .ti. OR tool* .ab. | 1.167.167 |
| --- | --- | --- |
| 16 | instrument* .ti. OR instrument* .ab. | 409.590 |
| 17 | "predictive model*" .ti. OR "predictive model*" .ab. | 33.985 |
| 18 | "prediction model*".ti. or "prediction model*".ab. | 37.543 |
| 19 | questionnaire* .ti. OR questionnaire* .ab. | 869.820 |
| 20 | "Risk assessment" .ti. OR "Risk assessment" .ab. | 96.581 |
| 21 | index* .ti. OR index* .ab. | 1.386.729 |
| 22 | inventor* .ti. OR inventor* .ab. | 141.044 |
| 23 | survey* .ti. OR survey* .ab. | 966.257 |
| 24 | "assessment method*" .ti. OR "assessment method*" .ab. | 23.345 |

| 25 | 13 or 14 or 15 or 16 or 17 or 18 or 19 or 20 or 21 or 22 or 23 or 24 | 5.011.896 |
| --- | --- | --- |

**Building block 3**

Emtree

| 26 | Exp aged/ | 3.369.744 |
| --- | --- | --- |
| 27 | Exp geriatrics/ | 39.816 |

Free terms

| 28 | aged.ti. or aged.ab. | 897.647 |
| --- | --- | --- |
| 29 | elde*.ti. or elde*.ab. | 403.913 |
| 30 | olde*.ti. or olde*.ab. | 723.383 |
| 31 | geriatric*.ti. or geriatric*.ab. | 85.479 |
| 32 | centenarian*.ti. or centenarian*.ab. | 2.658 |
| 33 | centarian*.ti. or centarian*.ab. | 3 |
| 34 | nonagenarian*.ti. or nonagenarian*.ab. | 2.272 |
| 35 | octogenarian*.ti. or octogenarian*.ab. | 5.873 |
| 36 | octagenarian*.ti. or octagenarian*.ab. | 108 |
| 37 | septuagenarian*.ti. or septuagenarian*.ab. | 634 |
| 38 | "very old".ti. or "very old".ab. | 6.340 |
| 39 | senior*.ti. or senior*.ab. | 66.212 |

| 40 | 26 or 27 or 28 or 29 or 30 or 31 or 32 or 33 or 34 or 35 or 36 or 37 or 38 or 39 | 4.466.049 |
| --- | --- | --- |

**Building block 4**

Free terms

| 41 | "risk factor*".ti. or "risk factor*".ab. | 1.015.782 |
| --- | --- | --- |
| 42 | variable*.ti. or variable*.ab. | 1.259.573 |
| 43 | predictor*.ti. or predictor*.ab | 682.823 |
| 44 | parameter*.ti. or parameter*.ab. | 1.548.059 |
| 45 | deficit*.ti. or deficit*.ab. | 367.583 |
| 46 | characteristic*.ti. or characteristic*.ab. | 2.120.265 |
| 47 | determinant*.ti. or determinant*.ab. | 319.672 |
| 48 | criteri*.ti. or criteri*.ab. | 1.188.805 |

| 49 | 41 or 42 or 43 or 44 or 45 or 46 or 47 or 48 | 6.919.451 |
| --- | --- | --- |

**Building blocks combined (exploratory search, date: 27-May-2022)**

| 12 and 25 and 40 and 49 | 29.344  (no filters used) |
| --- | --- |
| 12 and 25 and 40 and 49 **filter: limit to (embase and (dutch or english) and yr="2021 - current")** | 4.400 (including approximately 268 reviews identified using filter: Review) |
| 12 and 25 and 40 and 49 **filter: limit to (embase and (dutch or english) and yr="2018 - 2020" and aged <65+ years>)** | 1.747(including approximately 46 reviews identified using filter: Review) |

**Inclusion criteria:**

1. Topic of article = Frailty

**Exclusion criteria:**

1. Frailty is the independent variable for another outcome and there is no description of independent variables predicting frailty. (Rationale: fits goal to find independent variables to predict frailty.)(e.g. Research article describing how much frailty predicts complications or mortality without describing which variables predict frailty = exclusion)
2. Article type: case studies are excluded. (Rationale: we consider case studies not suitable for providing information on potential predictor variables.)
3. Full text not available in English / Dutch language (Rationale: readability, no full text article = no complete review possible.)

**Web of Science**

**Date: 31-Aug-2022**

**Building block 1**

Topics

| 1 | TS=(frail elderly) | 8.874 |
| --- | --- | --- |
| 2 | TS=(frailty) | 30.655 |
| 3 | TS=(functional status) | 86.075 |

Synonyms:

| 4 | TI=(frail*) OR AB=(frail*) | 34.169 |
| --- | --- | --- |
| 5 | TI=(debilit*) OR AB=(debilit*) | 27.509 |
| 6 | TI=(geriatric syndrome*) OR AB=(geriatric syndrome*) | 3.177 |

Free terms:

| 7 | TI=(pre-frail*) OR AB=(pre-frail*) | 1.251 |
| --- | --- | --- |
| 8 | TI=(functional status) OR AB=(functional status) | 69.071 |
| 9 | **TI=(fragil*) OR AB=(fragil*)** | 73.376 |
| 10 | TI=vulnerab* OR AB=vulnerab* | 281.834 |

Possible antonyms:

| 11 | TI=(resilien*) OR AB=(resilien*) | 128.626 |
| --- | --- | --- |

| 12 | 1 or 2 or 3 or 4 or 5 or 6 or 7 or 8 or 9 or 10 or 11 | 613.764 |
| --- | --- | --- |

**Building block 2**

Topics

| 13 | TS=(Risk Assessment) | 445.219 |
| --- | --- | --- |
| 14 | TS=(questionnaire) or TS=(health survey) | 1.024.271 |
| 15 | TS=(sensitivity and specificity) | 258.144 |

Free terms

| 16 | TI=(tool*) OR AB=(tool*) | 1.549.734 |
| --- | --- | --- |
| 17 | TI=(instrument*) OR AB=(instrument*) | 574.514 |
| 18 | TI=(predictive model*) OR AB=(predictive model*) | 192.688 |
| 19 | TI=(prediction model*) OR AB=(prediction model*) | 545.575 |
| 20 | TI=(questionnaire*) OR AB=(questionnaire*) | 684.710 |
| 21 | TI=(risk assesment) OR AB=(risk assesment) | 195 |
| 22 | TI=(index*) OR AB=(index*) | 1.415.752 |
| 23 | TI=(inventor*) OR AB=(inventor*) | 180.962 |
| 24 | TI=(survey*) OR AB=(survey*) | 1.237.710 |
| 25 | TI=(assessment method*) OR AB=(assessment method*) | 681.443 |

| 26 | 13 or 14 or 15 or 16 or 17 or 18 or 19 or 20 or 21 or 22 or 23 or 24 or 25 | 6.341.045 |
| --- | --- | --- |

**Building block 3**

Topics

| 27 | TS=(aged) | 3.563.094 |
| --- | --- | --- |
| 28 | TS=(geriatrics) | 10.726 |

Free terms

| 29 | TI=(aged) OR AB=(aged) | 3.397.481 |
| --- | --- | --- |
| 30 | TI=(elde*) OR AB=(elde*) | 286.176 |
| 31 | TI=(olde*) OR AB=(olde*) | 605.259 |
| 32 | TI=(geriatric*) OR AB=(geriatric*) | 50.007 |
| 33 | TI=(centenarian*) OR AB=(centenarian*) | 2.618 |
| 34 | TI=(centarian*) OR AB=(centarian*) | 2 |
| 35 | TI=(nonagenarian*) OR AB=(nonagenarian*) | 1.857 |
| 36 | TI=(octogenarian*) OR AB=(octogenarian*) | 4.424 |
| 37 | TI=(octagenarian*) OR AB=(octagenarian*) | 67 |
| 38 | TI=(septuagenarian*) OR AB=(septuagenarian*) | 518 |
| 39 | TI=(very old) OR AB=(very old) | 96.256 |
| 40 | TI=(senior*) OR AB=(senior*) | 64.855 |

| 41 | 27 or 28 or 29 or 30 or 31 or 32 or 33 or 34 or 35 or 36 or 37 or 38 or 39 or 40 | 4.030.075 |
| --- | --- | --- |

**Building block 4**

Free terms

| 42 | TI=(risk factor*) OR AB=(risk factor*) | 1.029.754 |
| --- | --- | --- |
| 43 | TI=(variable*) OR AB=(variable*) | 1.711.967 |
| 44 | TI=(predictor*) OR AB=(predictor*) | 570.039 |
| 45 | TI=(parameter*) OR AB=(parameter*) | 3.138.792 |
| 46 | TI=(deficit*) OR AB=(deficit*) | 305.328 |
| 47 | TI=(characteristic*) OR AB=(characteristic*) | 3.052.300 |
| 48 | TI=(determinant*) OR AB=(determinant*) | 339.708 |
| 49 | TI=(criteri*) OR AB=(criteri*) | 1.031.244 |

| 50 | 42 or 43 or 44 or 45 or 46 or 47 or 48 or 49 | 9.435.083 |
| --- | --- | --- |

**Building blocks combined (exploratory search, date: 27-May-2022)**

| 12 and 26 and 41 and 50 | 32.666  (no filters used) |
| --- | --- |
| 12 and 26 and 41 and 50 **AND (PY==("2018" OR "2019" OR "2020") OR "2021" OR "2022") AND LA==("ENGLISH" OR "DUTCH"))** | 15.861 (including approximately 1.064 reviews identified using filter: Review) |

**Inclusion criteria:**

1. Topic of article = Frailty

**Exclusion criteria:**

1. Frailty is the independent variable for another outcome and there is no description of independent variables predicting frailty. (Rationale: fits goal to find independent variables to predict frailty.)(e.g. Research article describing how much frailty predicts complications or mortality without describing which variables predict frailty = exclusion)
2. Article type: case studies are excluded. (Rationale: we consider case studies not suitable for providing information on potential predictor variables.)
3. Full text not available in English / Dutch language (Rationale: readability, no full text article = no complete review possible.)
