# Supplementary material for: Registered report protocol: A scoping review to identify potential predictors as features for developing automated estimation of the probability of being frail in secondary care
Source: PLoS One. 2022 Sep 27;17(9):e0275230. doi: 10.1371/journal.pone.0275230 (PMC9514620; doi:10.1371/journal.pone.0275230)
Supplement: Attachment — Submitted filename: response_to_reviewers.docx [file pone.0275230.s005.docx]

**Response to reviewers**

PONE-D-21-38902R1

Registered report protocol: a scoping review to identify potential predictors as features for automated prediction of the risk of frailty in secondary care

PLOS ONE

*Dear editor, dear reviewers,*

*Thank you again for the useful comments. Hereby we submit our second revision of the manuscript. Our revisions can be found in the tracked changes in the manuscript. Our response to the comments and suggestions made by the editor and reviewers can be found below, in italics. Thank you for your consideration of these responses and revisions.*

**Response to the editor**

Additional Editor Comments:

I have assessed the reviewers’ comments and revised the new version of the manuscript myself. Unfortunately, there are still some major methodological concerns that must be addressed before the manuscript can be accepted as a registered protocol.

Editor 1a. I agree with reviewer #3 when they write that it is still unclear whether the authors wish to identify predictors of being frail or of becoming frail. This is a critical issue that was already present in the first round of reviews, when reviewers asked the authors to clarify whether they aimed to detect frail patients or to detect patients at risk of developing frailty. That lack of clarity is present in the authors’ response to my first comment, when they wrote that they “intend to carry out this review to gather input for building an automated tool to predict the risk of someone being frail, not to develop another frailty detection instrument”. Unfortunately, “a frailty detection instrument” can be defined as a tool to predict whether someone is frail. Hence, the authors decision to replace “frailty detection tool” with “tool for prediction of the risk of frailty” did not solve the problem raised in the first round of review.

*Response: We would like to clarify that it is our intention to predict the risk of someone being frail, not the risk of someone becoming frail. We do this with the objective to support healthcare professionals in making choices in diagnosing and treating patients’ health problems while considering the possible presence of frailty. The outcome of our future algorithm will be an estimation of the probability that someone is frail. Above a certain cut-off in this probability estimation (to be determined in a future study in collaboration with the domain experts), these patients can then be referred to the department of geriatrics for detecting whether frailty is indeed present, and for subsequent frailty management.*

*To clarify this we made some changes at several points in the manuscript, most explicitly in the title: “Registered report protocol: a scoping review to identify potential predictors as features for developing automated estimation of the probability of being frail in secondary care.”; in line 33 of the revised manuscript with track changes: “We aim to identify potential predictors that could be used as features for modeling algorithms on the basis of routine hospital EHR data to incorporate in an automated tool for estimating the probability of being frail.”; in line 89* *of the revised manuscript with track changes: “**Therefore, the aim of our study is to perform a scoping review to build the foundation for the development of an automated tool for estimating the probability of being frail on the basis of routine health care data present in the EHR in secondary care, by identifying all potentially relevant features to test as potential predictors in a modeling effort based on machine learning.”; and in line 318 of the revised manuscript with track changes: “The extensive list of potential predictors of frailty provided by this study can be used as an evidence-based foundation for a modeling effort using machine learning to develop algorithms to be used for automated estimation of the probability of being frail based on hospital EHR variables recorded in routine care in the near future.”*

Editor 1b. The authors now stated that “the aim of our study is to perform a scoping review to build the foundation for the development of an automated prediction tool for the risk of frailty on the basis of routine health care data present in EHR in secondary care, by identifying all potentially relevant features to test as potential predictors in a modelling effort based on machine learning.”

To “predict the risk of frailty” is too vague because it can mean both identifying people who are frail at this moment, as well as to predict who will develop frailty within a certain timeframe. Those remain two different goals that require different methods and justifications. For example, in the end of the introduction section the authors argued that their review will differ from two previous reviews on this subject because they “extracted variables from frailty instruments” whereas the authors aim to “identify all potentially relevant predictors for frailty that can be extracted from the hospital EHR, not limited to frailty instruments only”. Considering that most Frailty Indices were constructed based on EHR, it is likely that the results of this review will also include many variables already used to construct those indices and that most of their results will derive from those studies. Hence, unless the authors’ aim to predict the risk of people becoming frail in the future, it is unclear why the results of the two previous reviews cannot be used to feed their future study using machine learning.

*Response: As mentioned in the response on comment 1a of the Editor, it is our intention to estimate the probability of someone being frail, not the risk of someone becoming frail. We do this with the objective to support healthcare professionals in making choices in diagnosing and treating patients’ health problems while considering the possible presence of frailty. The outcome of our future algorithm will be an estimation of the probability that someone is frail. Above a certain cut-off in this probability estimation (to be determined in a future study in collaboration with the domain experts), these patients can then be referred to the department of geriatrics for detecting whether frailty is indeed present, and for subsequent frailty management.*

*We agree that it is likely that the results of our review will also include many variables derived from studies into the construction of frailty indices. However, with our search strategy we also hope to derive a considerable number of variables from other types of research which will complement our search for potential predictors to add as features in our machine learning efforts. Both mentioned review articles (Bouillon et al. 2013, searched since inception until 2011; Bery et al. 2020, searched since 2011 until March 2018) (and the articles included therein) will now be the starting point of our search, consistent with our response to the further points (editor point 3; reviewer #2, point 1) regarding the search strategy and data saturation approach, in response to which we changed our search strategy.*

*We changed our manuscript on this point, most explicitly in line 41 of the revised manuscript with track changes: “Second, we add two published literature reviews (and the articles included therein) [Bery 2020; Bouillon, 2013] to our search results. In these reviews, articles on potential predictor variables in frailty screening tools were included from inception until March 2018.”; in line 100 of the revised manuscript with track changes: “Therefore, we will perform an extensive search in a broad corpus of published literature since 2018. The earlier mentioned reviews (and the articles included therein) will then be added to our search results [8,13]. We strive to identify all potentially relevant predictors for the presence of frailty that can be extracted from the hospital EHR, not limited to frailty instruments only.”; in line 154 of the revised manuscript with track changes: “The review articles of Bery (2020) and Bouillon (2013) (and the articles included therein) will be added to our search results [8,13]. The goal is to identify and extract all potential predictors of the presence of frailty.”; and by deleting lines 164 from the revised manuscript with track changes: “~~Searching is continued until data saturation is reached. Data saturation is considered to have been reached when no new possible predictors can be found in the included primary research articles anymore. After the analysis of primary research articles from the first run (published ≥2018), the review articles from that run will be analyzed, starting with the most recent one. As review articles include earlier published literature, the search will be further extended if new possible predictors are found in the review articles, adding one year to the PubMed (MEDLINE), CINAHL Plus, and Embase search strategies in each new run until data saturation has been reached (i.e. the second run searching articles published in 2017 and check for data saturation, the third run searching articles published in 2016 and check for data saturation, and so on, including the process with review articles from the same search period each time). If no new possible predictors are found in the review articles in a run, data saturation is considered to be confirmed, and the search will close.~~”*

*Furthermore, we amended and updated figure 2 and the appendices 2 and 3 containing the search strategies.*

Editor 1c. The authors should also recognize that there are some very simple frailty detection tools available, such as the Study of Osteoporotic Fractures and the PRISMA-7 tools, which can be applied in less than four minutes by any healthcare professional.

*Response: We do recognize that there are several very simple frailty detection tools available, however, even though such a tool can be applied in less than four minutes, the amount of time is still considerable as it is nearly half of the total time a doctor has for a regular outpatient visit. Therefore, we believe an algorithm that gives an estimation of the probability of the presence of frailty in a patient is relevant, by increasing the number of frail patients that receive care adjusted to their needs.*

*We did not change our manuscript on this point.*

Editor 2. Reviewers have requested the authors to specify which kind of frailty definition they intend to adopt as a gold standard. For the purposes of the scoping review the authors intend to conduct, I understand that they may include studies that used different definitions of frailty. However, it is essential that they add a description to their methods explaining that when summarizing the literature, they will present the prediction variables according to the definition of frailty used in the original studies they were extracted from. I agree with the reviewers that, in the next phase of their research, when they will use artificial intelligence to develop a model to predict the presence of frailty, they will have to define upfront what kind of definition of frailty they will use as the outcome of their prediction models. However, I understand that the authors may choose not to make that decision at the present moment. The downside is that their review will probably be more difficult to perform than if they decided to restrict their inclusion criteria to predictors of a specific type of frailty.

*Response: We agree with the editor that not choosing a specific type of frailty for this review may result in a more difficult performance of the review, however, we do believe it will strengthen our future research if we currently make no restrictions for retrieving potential variables for our machine learning effort. Furthermore, using artificial intelligence in a large sample of patients will allow us to use variables retrieved from all types of frailty measurements at the same time as well as combine these with other variables retrieved from the experience of domain experts. In the supervised machine learning process, we will use the reported results of available frailty screening tool(s) in the patient’s EHR as outcome variable(s). Finally, we intend to compare the outcome of our developed algorithm (the estimated probability of the presence of frailty) with frailty measured by comprehensive geriatric assessment (CGA) (if available in the patient’s EHR) and/or consensus among (inter)national domain experts upon reviewing the patient’s EHR as ‘gold standard’.*

*We added the definition of frailty used in an article as variable to extract in table 1; in line 200 of the revised manuscript with track changes: “The metadata of the articles (i.e., author(s), year of publication, study name, study design, study setting, and study country), descriptive baseline variables (i.e., study population, age, sex, and number of subjects), and all unique potential predictors of being frail (including relevant information such as the potential predictor type and the definition of frailty used in the article a potential predictor was mentioned; Table 1) will be extracted and recorded in Microsoft Excel.”; and in line 222 of the revised manuscript with track changes: “The results will be described in an extensive categorized table of all unique potential predictors for being frail, including total count of articles in which a potential predictor was mentioned and other relevant information such as the potential predictor type and the definition of frailty used in the article where a potential predictor was mentioned.”.*

Editor 3. While reading the authors responses, it appears that there are some misconceptions about the differences between systematic reviews and scoping reviews. Scoping reviews usually have a broader focus than systematic reviews. For example, both systematic reviews and scoping reviews should strive to perform exhaustive searches of the literature. The data saturation approach proposed by the reviewers is not compatible with the methodological expectations of both kinds of reviews. The argument described by the authors stating that “Moreover, by not continuing the search after data saturation has been reached, we prevent an enormous amount of work that would not lead to any increase in the yield of our research” is not compatible with PLOS ONE’s expectations of high methodological standards. I also find that the justification not to search the Web of Science database because the authors are conducting a scoping review inappropriate.

*Response: As mentioned in the response on editor 1b, we understand that the data saturation approach does not meet PLOS ONE’s expectations of high methodological standards. Also considering point 1 of reviewer #2 on the achievability of this approach, we have changed our search strategy. We let go the data saturation approach. Considering the current point raised by the editor, both review articles mentioned in 1b (Bouillon et al. 2013, searched since inception until 2011; Bery et al. 2020, searched since 2011 until March 2018) (and the articles included therein) will now be the starting point of our search. From that point on (2018 until present time), we will perform our own literature search. We also included searching Web of Science in our new search strategy.*

*We changed the methods section of our manuscript on this point, most explicitly in line* *118 of the revised manuscript with track changes: “Four databases will be searched for eligible articles: PubMed (MEDLINE), CINAHL Plus, Embase, and Web of Science.”; in line 154 of the revised manuscript with track changes: “The review articles of Bery (2020) and Bouillon (2013) (and the articles included therein) will be added to our search results.”; in line 255 of the revised manuscript with track changes: “An exploratory search in PubMed (MEDLINE), CINAHL Plus, Embase, and Web of Science resulted in a total of 32.526 records (7.796 PubMed (MEDLINE), 2.722 CINAHL Plus, 6.147 Embase, and 15.861 Web of Science, these were not yet checked for duplicate records) (Fig 2). ”; and by removing the first limitation in line 306 of the revised manuscript with track changes: “~~First, the literature is not searched across all available databases, which could result in missing articles and therefore potential predictors. In our opinion, the impact of this limitation is small, as we expect considerable overlap in the described potential predictors between the published articles~~”.*

*Furthermore, we amended and updated figure 2 and the appendices 2 and 3 containing the search strategies.*

Editor 4. The “synthesis of results” section does not provide sufficient information regarding how the author intend to report their results. “The results will be described in an extensive categorized table of all unique potential predictors for frailty, including total count of articles in which a potential predictor was

mentioned and other relevant information.” Specifically, what kind of “other relevant information” do the authors can anticipate to include in those tables? Will they take into account what kind of reference standard was used to define frailty? Will they describe the population of patients that was used in each study?

*Response: We agree with the editor that ‘other relevant information’ is a bit vague. The reason we kept this open is that we do not want to limit the way we report our synthesis beforehand. Furthermore, the essence of the machine learning approach in our future research is to use as many potential predictor variables as possible as features. The variables with the least predictive value will then automatically be dropped in the feature selection process.*

*As earlier mentioned in the response to comment 2 of the Editor, we added the definition of frailty used in an article as variable to extract in the methods section in line 200 of the revised manuscript with track changes: “The metadata of the articles (i.e., author(s), year of publication, study name, study design, study setting, and study country), descriptive baseline variables (i.e., study population, age, sex, and number of subjects), and all unique potential predictors of being frail (including relevant information such as the potential predictor type and the definition of frailty used in the article a potential predictor was mentioned; Table 1) will be extracted and recorded in Microsoft Excel.”; and in line 222 of the revised manuscript with track changes: “The results will be described in an extensive categorized table of all unique potential predictors of being frail, including total count of articles in which a potential predictor was mentioned and other relevant information such as the potential predictor type and the definition of frailty used in the article where a potential predictor was mentioned.”*

Editor 5. Lines 104 to 105: “Eligible articles are written in English or Dutch and should have an available full text to enable complete review.” That sentence remains inappropriate even after the authors response. If a reference cannot be retrieved by the Dutch Royal Library or from the authors, there is still no reason to say that it was not eligible for the review. It would be more accurate to simply explain that it was not retrieved, as expected by the recommendations of the PRISMA 2020 statement.

*Response: We agree with the editor that it would be more accurate to mention articles as not retrieved instead of not eligible in case full texts cannot be retrieved.*

*We changed our manuscript on this point in line 120 of the revised manuscript with track changes:* “*Articles that we were not able to get hold of as full-text will be reported as not retrieved.”*

Reviewer #1: I would like to thank the authors for the detailed response and clarifications. The manuscript has much improved and the remaining issues are out of the scope of this paper.

*Response: We would like to thank reviewer #1 for his positive reaction on our previous response.*

1) One final comment: while I understand that the aim of the study is to identify predictors of frailty in EHR data, it is still not entirely clear to me how “frailty” is defined in this context (e.g., whether it is considered as a deficit-accumulated frailty index, phenotypic frailty, or clinical frailty). Especially when using machine learning methods in the authors’ future work, which outcome/measure will be used as the reference standard to define frailty? This may perhaps be less relevant to the current protocol for the scoping review, but I encourage the authors to think about it and define more clearly on what they mean by “frailty” in their work.

*Response: In the supervised machine learning process, we will use the available reported results of frailty screening tool(s) in the patient’s EHR as outcome variable(s). Finally, we intend to compare the outcome of our developed algorithm (the estimated probability of the presence frailty) with frailty measured by comprehensive geriatric assessment (CGA) (if available in the patient’s EHR) and/or consensus among (inter)national domain experts upon reviewing the patient’s EHR as ‘gold standard’.*

*We added this to line 212 of the revised manuscript with track changes: “In the* *supervised machine learning methods, frailty will be defined as frailty present in steady state measured by the available reported results of frailty screening tools in the patient’s EHR. Finally, we intend to compare the outcome of the developed algorithm with frailty measured by comprehensive geriatric assessment (CGA)* *(if available in the patient’s EHR) and/or consensus among (inter)national domain experts upon reviewing the patient’s EHR as ‘gold standard.”.*

Reviewer #2:

Based on the changes to the manuscript, I now better understand the rationale for this work, which is to identify "predictors of frailty" which will then be fed into a machine learning algorithm and used on real patient data.

*Response: We would like to thank reviewer #2 for his/her additional comments and questions and for acknowledging that the rationale of our manuscript is now more understandable.*

However, I still have some concerns about the proposed methodology.

1) "Data saturation" --> having had personal experience with extracting frailty predictors from the literature, the volume of articles is incredible, and increases each year. This is especially true of articles deriving FIs based on the accumulation of deficits approach. I do not think saturation will be reached by the authors' current definition. There will always be one more FI that has a few more variables. I suggest the authors revise their plan to end the search

*Response: As mentioned in our response to editor’s points 1b and 3, we have changed our search strategy. We let go the data saturation approach. Both review articles mentioned in the response to comment 1b of the editor (Bouillon et al. 2013, searched since inception until 2011; Bery et al. 2020, searched since 2011 until March 2018) (and the articles included therein) will now be the starting point of our search. From that point on (March 2018 until present time), we will perform our own literature search.*

*We changed our manuscript on this point, most explicitly in line 154 of the revised manuscript with track changes: “The review articles of Bery (2020) and Bouillon (2013) (and the articles included therein) will be added to our search results [8,13].”; and by deleting lines 164 of the revised manuscript with track changes: “~~Searching is continued until data saturation is reached. Data saturation is considered to have been reached when no new possible predictors can be found in the included primary research articles anymore. After the analysis of primary research articles from the first run (published ≥2018), the review articles from that run will be analyzed, starting with the most recent one. As review articles include earlier published literature, the search will be further extended if new possible predictors are found in the review articles, adding one year to the PubMed (MEDLINE), CINAHL Plus, and Embase search strategies in each new run until data saturation has been reached (i.e. the second run searching articles published in 2017 and check for data saturation, the third run searching articles published in 2016 and check for data saturation, and so on, including the process with review articles from the same search period each time). If no new possible predictors are found in the review articles in a run, data saturation is considered to be confirmed, and the search will close.~~”*

*Furthermore, we amended and updated figure 2 and the appendices 2 and 3 containing the search strategies.*

2) Definition of types of articles that will be found in the search --> the authors should better define the kinds of articles they are hoping their search will find. Right now it is a bit vague. It is not just frailty measures, but quite a heterogeneous groups of articles on older people and assessment. In plain English under "literature search" they should spell this out

*Response: As shown in figure 1, we did explain the selection criteria in detail. As mentioned before in our response to comments 1b and 2 of the Editor, it is indeed likely that the results of our review will also include many variables derived from studies into the construction of frailty indices. However, with our search strategy we also want to derive a considerable number of variables from other types of research which will complement our search for potential predictors to add as features in our machine learning efforts. Making no restrictions for retrieving potential variables (based on a specific type of frailty or frailty measurement) will strengthen our future research. The articles we look for are all those about elderly patients in which frailty is described as dependent variable and in which the independent variables for frailty are described. We expect most results will be quantitative, observational studies. We do not want to include case studies.*

*We did not change our manuscript on this point.*

Reviewer #3:

1) What led your team to choose a scoping review over a systematic review? The field of frailty is not new, and it seems that the team is ready to do a systematic review to inform research, rather than a scoping review to describe the state of the field...

*Response: Indeed, the field of frailty is not new. However, Munn et al. (Systematic review or scoping review? Guidance for authors when choosing between a systematic or scoping review approach (2018), doi:* [*https://doi.org/10.1186/s12874-018-0611-x*](https://doi.org/10.1186/s12874-018-0611-x)*) state that one of the indications for performing a scoping review is to identify key characteristics or factors related to a concept. As it is our aim to identify as many factors related to the concept of (the presence of) frailty as possible – to be used as features in our machine learning efforts – we do believe that a scoping review approach is valid.*

*We did not change our manuscript on this point.*

2) If you are developing a prediction tool for frailty, rather than a frailty index, then you will need to choose your "gold standard" definition of frailty. How do you intend to do so? using which definition? how do you separate out the elements that define frailty (deficits in n index, features in FRAIL scale or Fried's phenotype) vs. upstream predictors of future frailty?

*Response: As mentioned in our responses to editor’s point 2 and reviewer #1, we will use the available reported results of frailty screening tool(s) in the patient’s EHR as outcome variable(s) in the supervised machine learning process. Finally, we intend to compare the outcome of our developed algorithm (the estimated probability of frailty) with frailty measured by comprehensive geriatric assessment (CGA) (if available in the patient’s EHR) and/or consensus among (inter)national domain experts upon reviewing the patient’s EHR as ‘gold standard’.*

*We added this to line 212 of the revised manuscript with track changes: “In the supervised machine learning methods, frailty will be defined as frailty present in steady state measured by the available reported results of frailty screening tools in the patient’s EHR. Finally, we intend to compare the outcome of the developed algorithm with frailty measured by comprehensive geriatric assessment (CGA) (if available in the patient’s EHR) and/or consensus among (inter)national domain experts upon reviewing the patient’s EHR as ‘gold standard”.*

3) Again, in reviewing response to questions - are you differentiating between someone BEING frail vs. BECOMING frail at a point in the future? How are you defining whether frailty is "present in steady state"?

*Response: As mentioned in our response to editor’s point 1a, we would like to clarify that it is our intention to estimate the probability of someone being frail, not of someone becoming frail. We do this with the objective to support healthcare professionals in making choices in diagnosing and treating patients’ health problems while considering the possible presence of frailty. The outcome of our future algorithm will be an estimation of the probability that someone is frail. Above a certain cut-off in this probability estimation (to be determined in a future study in collaboration with the domain experts), these patients can then be referred to the department of geriatrics for detecting whether frailty is indeed present, and for subsequent frailty management.*

*To clarify this we made some changes at several points in the manuscript, most explicitly in the title: “Registered report protocol: a scoping review to identify potential predictors as features for developing automated estimation of the probability of being frail in secondary care.”; in line 33 of the revised manuscript with track changes: “We aim to identify potential predictors that could be used as features for modeling algorithms on the basis of routine hospital EHR data to incorporate in an automated tool for estimating the probability of being frail.”; in line 89 of the revised manuscript with track changes: “Therefore, the aim of our study is to perform a scoping review to build the foundation for the development of an automated tool for estimating the probability of being frail on the basis of routine health care data present in the EHR in secondary care, by identifying all potentially relevant features to test as potential predictors in a modeling effort based on machine learning.”; and in line 318 of the revised manuscript with track changes: “The extensive list of potential predictors of frailty provided by this study can be used as an evidence-based foundation for a modeling effort using machine learning to develop algorithms to be used for automated estimation of the probability of being frail based on hospital EHR variables recorded in routine care in the near future.”*
